# Systemic Neutralization of Granulocyte–Macrophage Colony-Stimulating Factor Attenuates Stellate Ganglion Neuroinflammation and Cardiac Sympathetic Overactivation in Chronic Heart Failure in Rats

**DOI:** 10.3390/ijms27156876

**Published:** 2026-07-31

**Authors:** Sulail Fatima, Lauren Whitney, Yu Li, Boris Shabaltiy, Yu-Long Li

**Affiliations:** Department of Emergency Medicine, University of Nebraska Medical Center, Omaha, NE 68150-5850, USA; sfatima@unmc.edu (S.F.);

**Keywords:** chronic heart failure, GM-CSF, neuroinflammation, stellate ganglion, sympathetic overactivation

## Abstract

Chronic heart failure (CHF) is characterized by heightened cardiac sympathetic activity and neuroinflammation within the stellate ganglia (SG), contributing to cardiac arrhythmogenesis. However, the upstream mediators of this neuroimmune interaction remain incompletely defined. This study investigated whether systemic neutralization of granulocyte–macrophage colony-stimulating factor (GM-CSF, an inflammatory cytokine) attenuates SG inflammation and cardiac sympathetic overactivation in a rat model of coronary artery ligation-induced CHF. Male *Sprague–Dawley* rats underwent myocardial infarction or sham surgery and were treated with a GM-CSF neutralizing antibody or vehicle. Cardiac sympathetic nerve activity (CSNA), heart rate variability (HRV), cytokine expression, and the electrophysiological properties of cardiac sympathetic post-ganglionic (CSP) neurons were assessed. CHF increased the levels of GM-CSF, ionized calcium-binding adaptor molecule 1 (IBA1, an activated macrophage marker) and pro-inflammatory markers (TNF-α, pro IL-1β) in the SG, enhanced neuronal Ca^2+^ currents, cell excitability, and CSNA, and impaired HRV. The GM-CSF neutralizing antibody significantly attenuated SG inflammation, partially normalized Ca^2+^ currents and neuronal firing, decreased CSNA, and improved cardiac autonomic balance. These findings identify GM-CSF as a key regulator of SG neuroinflammation and sympathetic dysregulation in CHF. Targeting this GM-CSF protein may represent a novel therapeutic strategy to mitigate autonomic imbalance and arrhythmic risk.

## 1. Introduction

Heart failure (HF) remains a prominent global health problem, currently affecting over 60 million individuals and serving as a primary driver of cardiovascular morbidity and mortality [1]. A critical challenge in the clinical care of HF is the high incidence of sudden cardiac death (SCD), which accounts for nearly half of all HF-related fatalities [2]. This lethal susceptibility stems from development of arrhythmogenic substrates, where adverse myocardial structural and electrophysiological remodeling converges with persistent cardiac sympathetic overactivation [3,4]. Excess sympathetic activation not only causes hemodynamic stress but also promotes ventricular arrhythmias by increasing abnormal afterdepolarizations, accelerating repolarization, and creating regional differences in electrical activity across the myocardium [5,6]. Despite the implementation of guideline-directed medical therapy and advancements in device-based interventions, the electrical instability inherent to the failing heart is not fully suppressed [7]. Consequently, there is an urgent need to elucidate the upstream neural mechanisms that lower the threshold for malignant ventricular arrhythmias and identify novel therapeutic targets within the autonomic axis.

The stellate ganglia (SG) are essential regulatory centers for cardiac sympathetic control providing post-ganglionic innervation to the myocardium and the cardiac conduction system [3]. Under physiological conditions, cardiac function relies on the tightly regulated release of norepinephrine (NE) from post-ganglionic sympathetic nerve terminals. In chronic heart failure (CHF), however, this homeostatic control is fundamentally disturbed, leading to a maladaptive sympathetic surge [8]. Pathological remodeling of the SG in CHF involves both immune cell-derived cytokine accumulation and altered ion channel dysfunction, a combination that heightens neuronal excitability and sympathetic output [9,10,11]. In parallel, preclinical data suggest that macrophage expansion within the SG directly enhances neuronal excitability and promotes cardiac sympathetic overactivation and ventricular arrhythmogenesis [9,12]. Recent work also demonstrates that attenuation of macrophage activation and neuroinflammation within the SG, such as through renal denervation or macrophage depletion, can reduce sympathetic outflow and suppress arrhythmic events in CHF [9,13].

Granulocyte–macrophage colony-stimulating factor (GM-CSF) is a pleiotropic cytokine that governs the proliferation, survival, and polarization of monocyte-lineage cells [14]. GM-CSF also acts as a potent pro-inflammatory orchestrator by driving the functional reprogramming of macrophages towards a highly active state characterized by increased oxidative stress and sustained IL-1β production [15,16,17]. Clinically, circulating GM-CSF levels are significantly elevated in CHF patients and demonstrate a positive correlation with disease severity, reduced ejection fraction, and elevated plasma norepinephrine levels [18]. Our laboratory recently found that GM-CSF and its receptor-α are elevated in the SG in CHF and that renal denervation reduces GM-CSF along with macrophage activation and neuroinflammation in the SG [13]. Because renal denervation reduces GM-CSF as one of several downstream effects, the study could not establish whether GM-CSF itself is sufficient to drive SG neuroinflammation or whether directly neutralizing GM-CSF is protective [13]. Therefore, we hypothesized that GM-CSF facilitates the recruitment and activation of macrophages within the SG, establishing a neuroinflammatory milieu. This inflammatory state, in turn, triggers pathological ionic remodeling in cardiac sympathetic post-ganglionic (CSP) neurons, thereby sustaining sympathetic overactivation in CHF. Given that GM-CSF is a circulating cytokine, we examined the effect of systemic GM-CSF neutralization on SG neuroinflammation, CSP neuronal excitability, cardiac sympathetic nerve activity (CSNA), and heart rate variability (HRV) in a rat model of CHF.

## 2. Results

### 2.1. Confirmation of CHF Phenotype and Effect of GM-CSF Neutralization on CHF Phenotype

Echocardiography was performed at 12 weeks post-MI or sham treatment in sham, CHF alone, and CHF rats before receiving GM-CSF neutralization (Supplementary Figure S1A–C and Table S1). Compared with the sham group, both CHF groups showed significant reductions in ejection fraction and fractional shortening and increases in dimensions during systole and diastole, confirming an established heart failure phenotype in CHF animals (*p* < 0.05 vs. sham). In addition, we applied a paired *t*-test to determine the effect of GM-CSF neutralization on the CHF phenotype. There were no significant differences in cardiac function and dimensions between before (12 weeks post-MI) and after (14 weeks post-MI) treatment with GM-CSF neutralization (Supplementary Figure S7C,D and Table S2).

### 2.2. Neutralization of GM-CSF Reduces Inflammatory Markers in Stellate Ganglia and Dampens Tissue-Specific GM-CSF Upregulation

**Figure 1:**
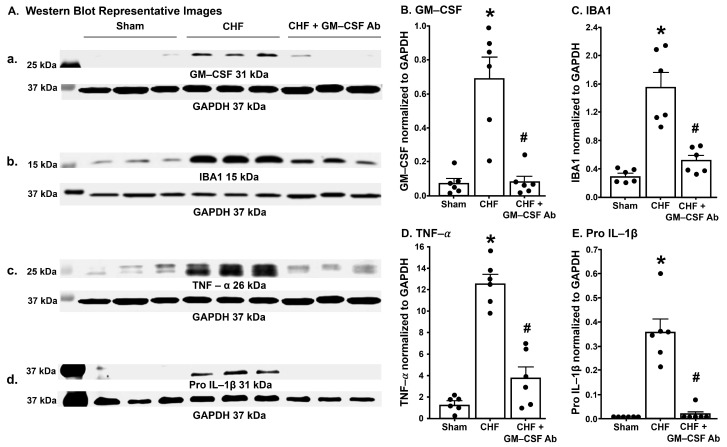
GM−CSF neutralization attenuates inflammatory marker expression in the stellate ganglia (SG) during CHF. (**A**) Representative immunoblots for (**a**) GM-CSF, (**b**) IBA1, (**c**) TNF-α, and (**d**) pro IL-1β in SG tissue lysates across sham, CHF, and CHF + GM-CSF Ab treatment groups. GAPDH was used as the internal loading control for all proteins. (**B**–**E**) Densitometric quantification of (**B**) GM-CSF, (**C**) IBA1, (**D**) TNF-α, and (**E**) pro IL-1β protein expression normalized to GAPDH. Data are expressed as mean ± SEM (*n* = 6 per group). Statistical significance was determined by one-way ANOVA with Tukey’s post hoc test or Kruskal–Wallis test followed by Dunn’s test where appropriate. * *p* < 0.05 vs. sham, ^#^ *p* < 0.05 vs. CHF.

**Figure 2:**
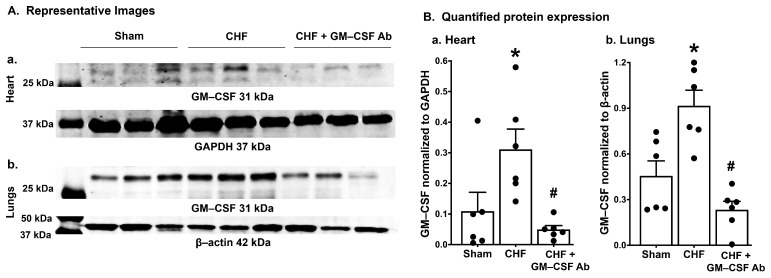
GM−CSF expression in the heart and lungs in all experimental groups. (**A**) Representative immunoblots of GM-CSF in (**a**) heart and (**b**) lung tissue lysates from sham, CHF, and CHF + GM-CSF Ab groups. GAPDH and β-actin served as internal loading controls for heart and lung samples, respectively. (**B**) Densitometric quantification of GM-CSF protein expression in (**a**) heart and (**b**) lung tissues. Heart data were analyzed using the Kruskal–Wallis test followed by Dunn’s post hoc test. Lung data were analyzed using one-way ANOVA followed by Tukey’s post hoc test. Data are presented as mean ± SEM (*n* = 6 per group). * *p* < 0.05 vs. sham, ^#^ *p* < 0.05 vs. CHF.

**Figure 3:**
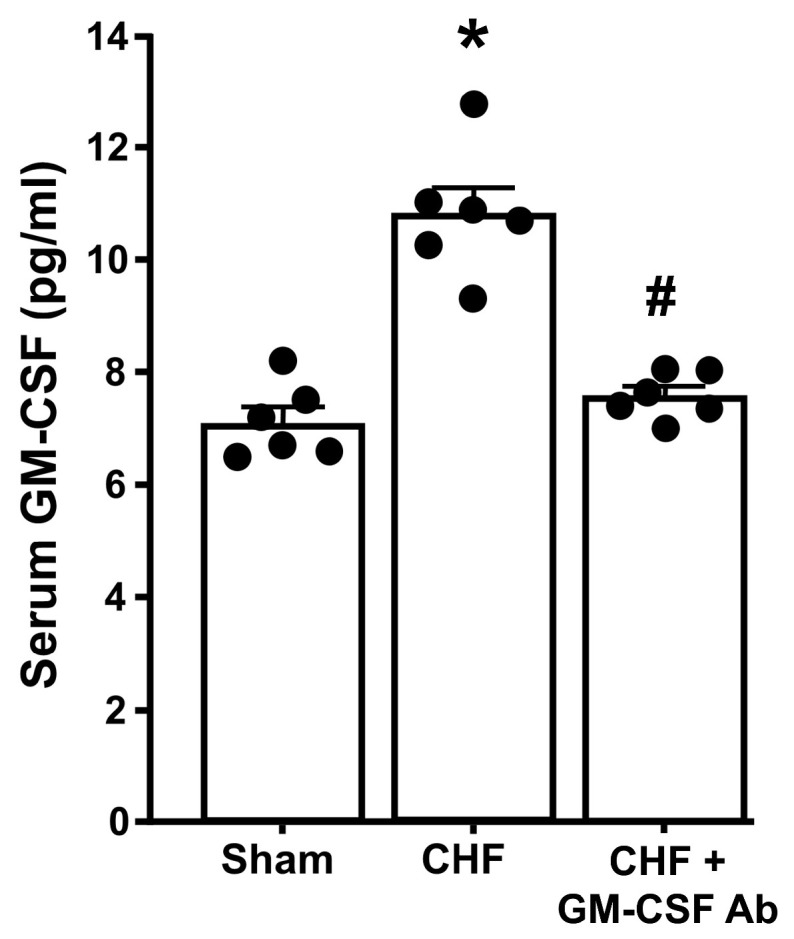
Systemic neutralization of GM−CSF reduces circulating GM−CSF levels in CHF rats. Serum GM-CSF concentrations (pg/mL) measured by ELISA in sham, CHF, and CHF + GM-CSF Ab groups. Data are presented as mean ± SEM (*n* = 6 per group). Statistical significance was evaluated using Welch’s ANOVA followed by Dunnett’s post hoc test for multiple comparisons. * *p* < 0.05 vs. sham, ^#^ *p* < 0.05 vs. CHF.

The induction of CHF resulted in a significant increase in GM-CSF protein expression within the SG (*p* = 0.012), accompanied by elevated levels of IBA1 (an activated macrophage marker, *p* = 0.006) and the pro-inflammatory markers TNF-α (*p* < 0.001) and Pro IL-1β (*p* = 0.003) in comparison to sham animals. In CHF rats, systemic neutralization of GM-CSF significantly reduced the expression of GM-CSF (*p* = 0.008), IBA1 (*p* = 0.01), TNF-α (*p* < 0.001) and Pro IL-1β (*p* = 0.028) within the SG (Figure 1). CHF also resulted in a marked elevation of GM-CSF levels in both the heart (*p* = 0.045) and lung tissues (*p* = 0.025), suggesting a state of localized inflammatory remodeling in response to the disease. However, in the CHF + GM-CSF Ab group, the expression of the GM-CSF protein was significantly reduced in both organs (heart: *p* = 0.028, lungs: *p* = 0.001) compared to the untreated CHF group (Figure 2). Additionally, we found that systemic neutralization significantly lowered CHF-enhanced circulating GM-CSF levels in the serum, reducing concentrations from 10.80 ± 0.47 pg/mL in the CHF group to 7.60 ± 0.17 pg/mL in the CHF + GM-CSF Ab group (*p* = 0.005 vs. CHF; Figure 3).

### 2.3. Effects of GM-CSF Ab on CHF-Altered Ca^2+^ Currents and Cell Excitability of CSP Neurons

**Figure 4:**
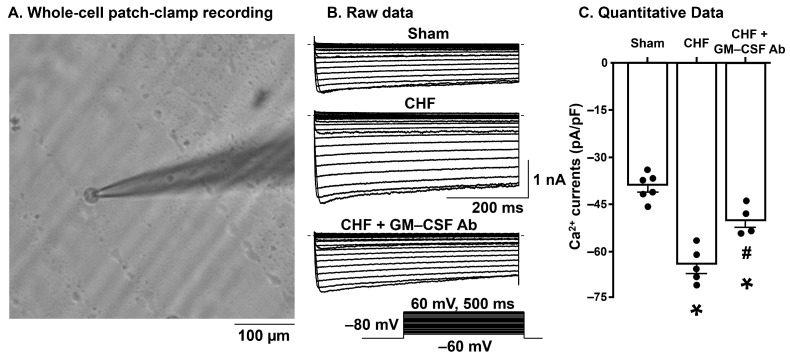
GM−CSF neutralization attenuates the increase in calcium current density in SG neurons during CHF. (**A**) Representative bright-field micrograph of a sympathetic post-ganglionic neuron from the SG during whole-cell patch-clamp recording. Scale bar = 100 μm. (**B**) Representative traces of voltage-gated Ca^2+^ currents recorded from sham, CHF, and CHF + GM−CSF antibody (Ab) groups. Currents were elicited by a voltage step protocol from a holding potential of −80 mV (−60 mV to +60 mV with 5 mV increments, 500 ms duration). (**C**) Quantitative analysis of mean Ca^2+^ current density (pA/pF). Data represent currents elicited by a 500 ms test pulse at 0 mV from a holding potential of −80 mV. Values are presented as mean ± SEM (*n* = 4–6 neurons per group). Statistical significance was determined by one-way ANOVA followed by Tukey’s post hoc test. * *p* < 0.05 vs. sham, ^#^ *p* < 0.05 vs. CHF.

**Figure 5:**
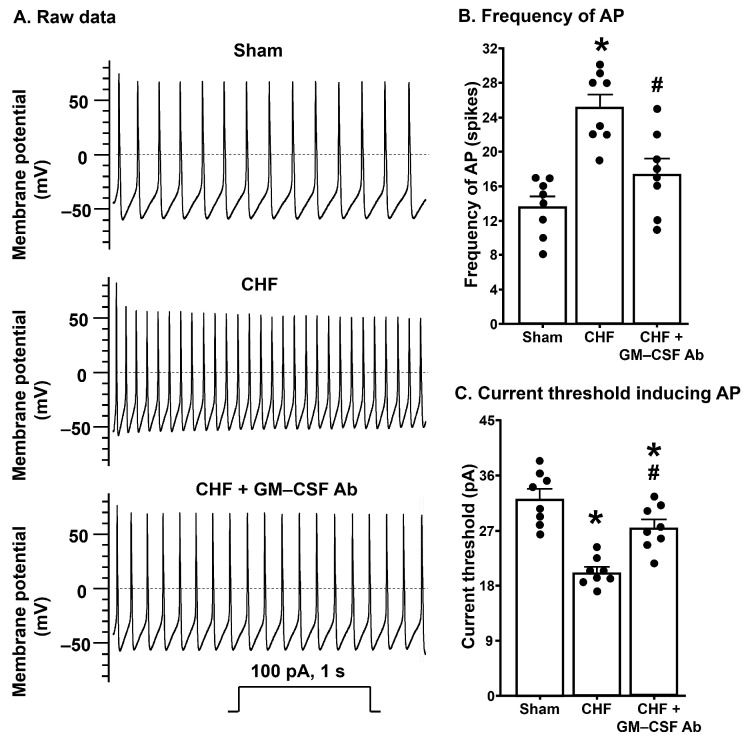
GM−CSF neutralization attenuates increased neuronal excitability in SG neurons of CHF rats. (**A**) Representative membrane potential tracings from sympathetic post-ganglionic neurons across the sham, CHF, and CHF + GM-CSF Ab groups. (**B**) Quantitative data for frequency of action potentials (spikes per second) and (**C**) current threshold (pA) required to elicit an action potential. Data are presented as mean ± SEM (*n* = 8 neurons per group). Statistical significance was evaluated using one-way ANOVA followed by Tukey’s post hoc test. * *p* < 0.05 vs. sham, ^#^ *p* < 0.05 vs. CHF.

Fourteen weeks post-MI, CSP neurons from CHF animals exhibited a significant increase in voltage-gated calcium currents, with peak current densities accelerating from −39.47 ± 1.71 pA/pF in the sham group to −64.58 ± 2.56 pA/pF in the CHF group (*p* < 0.001, Figure 4). This CHF-induced ionic remodeling was accompanied by a nearly double increase in neuronal cell excitability from 13.6 ± 1.18 Hz in the sham group to 25.1 ± 1.44 Hz in the CHF group (*p* < 0.001) and a sharp drop in the current threshold required to trigger an action potential (AP) from 32.2 ± 1.55 pA in sham animals to 20.2 ± 0.80 pA in the CHF animals (*p* < 0.001) (Figure 5). Systemic treatment with the GM-CSF neutralizing antibody successfully blunted this pathological phenotype induced by CHF. Antibody administration reduced the peak calcium current density to −49.97 ± 2.40 pA/pF (*p* = 0.002; Figure 4). Furthermore, GM-CSF blockade also decreased neuronal excitability, significantly elevating the activation threshold to 27.6 ± 1.29 pA (*p* = 0.001) and reducing AP firing frequency to 17.5 ± 1.66 Hz (*p* = 0.003; Figure 5).

### 2.4. GM-CSF Neutralization Reduces CHF-Induced Cardiac Sympathetic Nerve Overactivation

**Figure 6:**
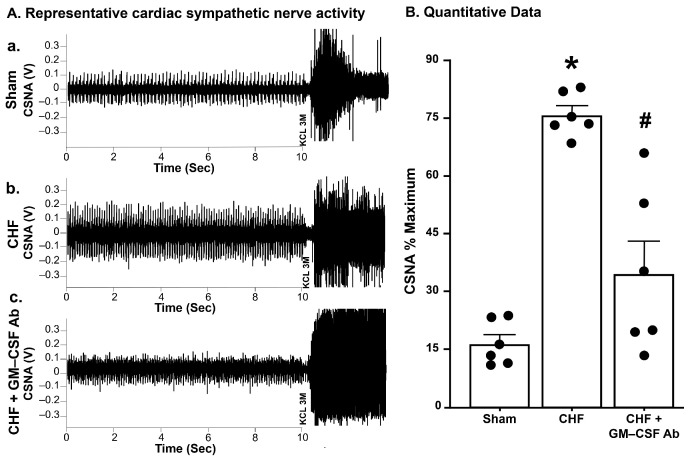
GM−CSF neutralization reduces the exaggerated CSNA in CHF rats. (**A**) Representative raw tracings of CSNA recorded at baseline and in maximum response to intracardiac KCl (3 M) injection across (**a**) sham, (**b**) CHF, and (**c**) CHF + GM-CSF Ab groups. (**B**) Quantitative analysis of CSNA, represented as a percentage of the maximum response (% Max). Data are expressed as mean ± SEM (*n* = 6 per group). Statistical significance was evaluated using Welch’s ANOVA followed by Dunnett’s post hoc test for multiple comparisons. * *p* < 0.05 vs. sham, ^#^ *p* < 0.05 vs. CHF.

To investigate the functional consequences of reduced neuroinflammation in the SG, we measured cardiac sympathetic nerve activity (CSNA) across all groups. CHF resulted in a marked increase in CSNA, driving nerve activity from 16.4 ± 2.36% of the maximum in the sham group to a pathological peak of 75.9 ± 2.28% of the maximum in the CHF group (*p* < 0.001, Figure 6). However, systemic administration of the GM-CSF neutralizing antibody significantly blunted this chronic sympathetic overflow. In the CHF + GM-CSF Ab group, cardiac sympathetic efferent discharge was reduced to 34.5 ± 8.60% of the maximum (*p* = 0.009 vs. the CHF group; Figure 6).

### 2.5. GM-CSF Neutralization Restores HRV in CHF Rats

**Figure 7:**
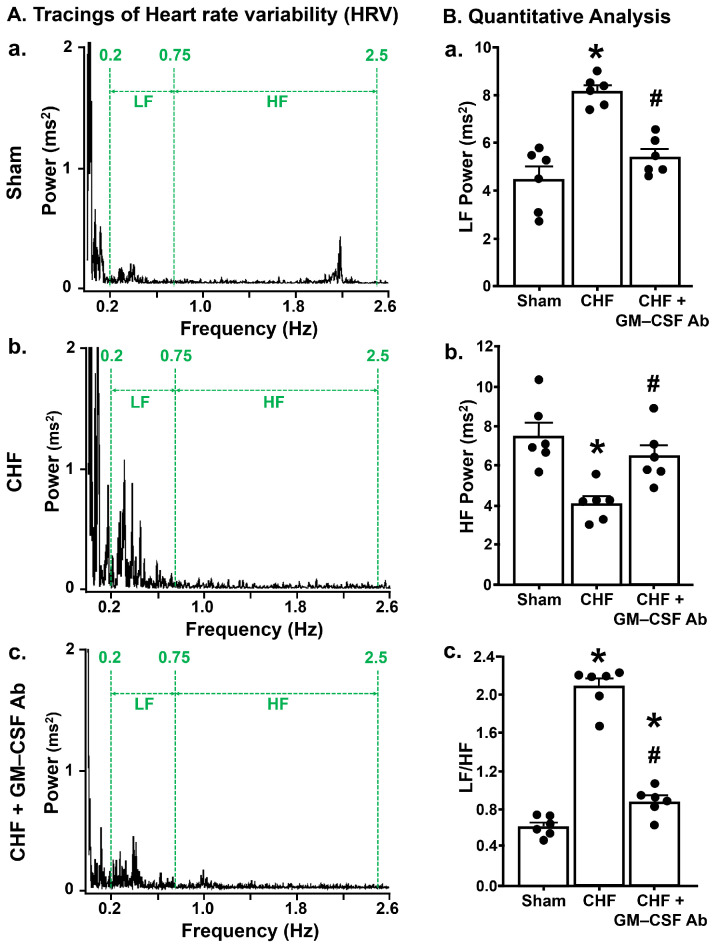
GM−CSF neutralization improves HRV in CHF rats. (**A**) Representative power spectral density tracings of HRV from (**a**) sham, (**b**) CHF, and (**c**) CHF + GM-CSF Ab groups. Dashed green lines delineate the low-frequency (LF: 0.2–0.75 Hz) and high-frequency (HF: 0.75–2.5 Hz) spectral bands. (**B**) Quantitative analysis of (**a**) LF power (ms^2^), (**b**) HF power (ms^2^), and (**c**) the calculated LF/HF ratio across all experimental groups. Data are presented as mean ± SEM (*n* = 6 per group). Statistical significance was evaluated using one-way ANOVA followed by Tukey’s post hoc test. * *p* < 0.05 vs. sham, ^#^ *p* < 0.05 vs. CHF.

To evaluate overall cardiac autonomic function, we analyzed HRV as a marker of sympathetic–parasympathetic balance (Figure 7). CHF resulted in a profound shift toward sympathetic dominance, evidenced by a significant increase in low-frequency (LF) power from 4.48 ± 0.53 ms^2^ in the sham group to 8.18 ± 0.24 ms^2^ in the CHF group (*p* < 0.001) and the LF/high-frequency (HF) ratio from 0.625 ± 0.042 in sham animals to 2.08 ± 0.09 in CHF animals (*p* < 0.001). Conversely, parasympathetic activity, represented by HF power, was significantly diminished in the CHF group, dropping from 7.53 ± 0.66 ms^2^ in sham animals to 4.10 ± 0.37 ms^2^ in CHF animals (*p* = 0.001). Consequently, the systemic neutralization of GM-CSF Ab significantly reversed this severe autonomic imbalance in CHF rats. In the CHF + GM-CSF Ab group, LF power and the LF/HF ratio were reduced to 5.43 ± 0.313 ms^2^ and 0. 883 ± 0.06, respectively (*p* < 0.001 vs. CHF), while HF power increased to 6.47 ± 0.57 ms^2^ (*p* = 0.02 vs. CHF).

## 3. Discussion

### 3.1. Major Findings

The present study identifies GM-CSF as an important regulator of the neuroimmune crosstalk within the SG that contributes to sympathetic overactivation in CHF. Our results demonstrate that systemic neutralization of GM-CSF attenuates SG neuroinflammation and blunts the pathological ionic remodeling of cardiac sympathetic neurons. Extending our laboratory’s prior work in which surgical renal denervation reduced SG macrophage activation and lowered GM-CSF as one of several downstream effects [13], the present study neutralizes the ligand directly, isolating GM-CSF as a contributing upstream driver. By disrupting the inflammatory axis, we improved cardiac autonomic balance, reflected by more stable CSNA and HRV.

### 3.2. Circulating and Local Sources of GM-CSF

Chronic hemodynamic and ischemic stress are established drivers of sterile, multiorgan inflammation [19,20]. We observed a significant elevation of circulating serum GM-CSF levels in CHF, which mirrors clinical data where elevated plasma GM-CSF concentrations correlate with symptomatic severity and severe left ventricular dysfunction [18]. Our data further demonstrates localized upregulation of the GM-CSF protein in the heart, lungs, and SG, indicating both systemic recruitment and tissue-level production. This pattern may reflect contributions from GM-CSF-producing T-helper cells, which are expanded in coronary disease [21]. Notably, this response appeared to be organ-specific, as no elevation of GM-CSF was detected in the brain, liver, and kidneys in CHF, suggesting that CHF establishes a specialized inflammatory network linking peripheral organs with sympathetic ganglia.

In the heart, the finding of increased GM-CSF protein expression aligns with established evidence identifying cardiac fibroblasts as a primary cellular source of this cytokine following myocardial infarction (MI). Resident fibroblasts act as sensors for the necrotic environment, responding to danger-associated molecular patterns (DAMPs) to initiate a local inflammatory cascade [22]. While previous reports characterized myocardial GM-CSF production as a transient acute-phase response that peaks within 24 h [22,23], the sustained elevation observed at 14 weeks post-MI in the current study (Figure 1, Figure 2 and Figure 3) suggests a transition to a chronic pathological drive. The ongoing ischemic and mechanical strain likely maintains fibroblasts in an activated secretory state [24,25], transforming the failing myocardium into a sustained source of myeloid-stimulating factors. As a central regulator of myelopoiesis and leukocyte mobilization, persistent GM-CSF production provides a mechanistic basis for continued macrophage recruitment beyond the acute phase of injury [26]. Beyond the heart, elevated pulmonary GM-CSF identifies the lungs as an active inflammatory reservoir in CHF. This extends clinical observations of Sikkeland et al. [27], showing that alveolar macrophages in CHF patients exhibit heightened expression of TNF-α, IL-6, and Endothelin-1. The localized upregulation of GM-CSF within the pulmonary and myocardial tissue beds likely contributes to monocyte recruitment and macrophage expansion, processes that have been implicated in myocardial calcium handling abnormalities and β-adrenergic signaling dysfunction [28].

### 3.3. GM-CSF as a Mediator of SG Inflammation and Autonomic Remodeling

As the primary nexus for cardiac sympathetic outflow, the SG plays a central role in post-MI sympathetic hyperactivity and arrhythmogenesis [29]. Both clinical and preclinical evidence consistently demonstrate a conserved neuroinflammatory signature in the SG during CHF, featuring immune cell infiltration and satellite glial cell activation [10]. Histological evidence from patients with severe arrhythmias further supports the presence of T-lymphocyte-mediated ganglionitis, suggesting that infiltrating immune cells may increase adrenergic output through paracrine signaling [30]. Although macrophage-driven neuronal cell excitability in the SG has been recognized in CHF, the upstream signals sustaining this persistent ganglionic neuroinflammation remain unclear. Our finding of elevated GM-CSF within the SG aligns with prior work from our laboratory showing that Iba1^+^ macrophages express GM-CSF receptors and serve as a local source of TNF-α and IL-1β [9,13]. These macrophage-driven cytokines may remodel Ca^2+^ currents in CSP neurons. In parallel, GM-CSF signaling in post-ganglionic sympathetic neurons may independently facilitate neural structural remodeling such as neurite outgrowth and nerve-terminal sprouting, leading to increased sympathetic outflow [31,32]. In addition to GM-CSF-driven expansion, the functional phenotype of macrophages within the stellate ganglion is likely influenced by the local neuroimmune microenvironment [33]. Neurons in the SG closely interact with satellite glial cells (SGCs), which together regulate ganglionic excitability and inflammatory signaling [34]. Emerging evidence suggests that SGCs can modulate sympathetic neuronal activity and contribute to local cytokine production, thereby shaping immune cell activation within the ganglion [3,35,36,37]. In the setting of CHF, these interactions may form a self-reinforcing loop in which macrophage-derived cytokines and neuronal activity sustain one another. Within this context, GM-CSF-driven macrophage expansion may amplify TNF-α and IL-1β production, reinforcing the inflammatory milieu and promoting neuronal hyperexcitability within the SG.

In the current study, we first provide direct evidence that targeting the GM-CSF axis attenuates SG neuroinflammation and improves autonomic balance. CSP neurons from HF animals displayed increased voltage-gated Ca^2+^ currents, associated with heightened cell excitability, including increased firing frequency and a reduced activation threshold. These findings are particularly significant because Ca^2+^ currents predominantly trigger neurotransmitter release from cardiac sympathetic nerve terminals [37,38]. Pathological increases in these currents have been linked to enhanced CSNA and ventricular arrhythmogenesis in CHF [38]. Moreover, this ionic remodeling is intimately coupled to the localized immune environment in the SG. Previous work from our laboratory has shown that depleting macrophages within the SG directly reduces N-type Ca^2+^ currents and neuronal cell excitability, confirming a causal role of immune cells in maintaining this phenotype [9]. Here, our results demonstrate that blocking the GM-CSF axis not only reduced the expansion of activated macrophages and the expression of pro-inflammatory cytokines but also attenuated the increased Ca^2+^ currents and cell excitability of CSP neurons. The nature of this CHF-induced ionic remodeling has been defined previously by our laboratory [39]. In CSP neurons of the SG, the rise in voltage-gated Ca^2+^ currents and cell excitability during CHF occurs even though the expression of Ca^2+^ channel mRNA and protein remains unchanged, indicating that the functional change in Ca^2+^ channels reflects a post-translational (for instance, phosphorylation) rather than transcriptional mechanism [39]. Here, it implies that the CHF-elevated GM-CSF signaling raises voltage-gated Ca^2+^ currents by modulating Ca^2+^ channels already presented at the membrane. The persistence of this altered state in dissociated neurons held in culture medium for several hours further indicates that it is stably maintained rather than dependent on continuous cytokine exposure. Additionally, GM-CSF neutralization-induced reversal of Ca^2+^ currents is consistent with the removal of an inflammatory input that had been sustaining the modified channel function. Additionally, another question is how systemic neutralization reduces the SG macrophage population. Because GM-CSF promotes myeloid cell production and mobilization [14], the GM-CSF antibody may limit monocyte recruitment to the SG and reduce local macrophage proliferation. The low IBA1 and cytokine levels in the SG are consistent with either action of the GM-CSF antibody or the fact that GM-CSF acts as the upstream mediator in either way. Separating its local and recruitment-based effects will be an important goal for future studies to use SG-targeted or cell-specific approaches.

In the CHF state, the heart is subjected to a profound shift toward sympathetic dominance, characterized by a significant increase in LF power and a marked elevation of the LF/HF ratio [40,41]. This autonomic imbalance is a primary predictor of malignant arrhythmias and sudden cardiac death in patients with severe heart failure [42]. In our study, the stabilization of CSNA following GM-CSF neutralization was accompanied by a recovery of cardiac autonomic balance, as demonstrated by the dual improvement of reduced LF power and LF/HF ratio (cardiac sympathetic activation) and simultaneously restored HF power (cardiac parasympathetic activation). This modulation of both autonomic arms may be mediated by two mechanisms. First, the recovery of parasympathetic tone may be a functional byproduct of reciprocal interactive inhibition. By attenuating the inflamed ganglionic microenvironment, GM-CSF neutralization blunted the excessive sympathetic drive, effectively removing the inhibitory brake on the parasympathetic system and permitting the recovery of baseline cardiovagal modulation. Second, a more direct influence on parasympathetic circuitry may also coexist. By lowering circulating and tissue GM-CSF, systemic neutralization could reduce inflammatory input to the peripheral parasympathetic pathway; however, we did not assess vagal signaling or cardiac parasympathetic circuits, and this possibility remains speculative.

Two-week neutralization with GM-CSF resulted in no significant changes in ejection fraction, fractional shortening, and left ventricular dimensions when compared with the data obtained before treatment with GM-CSF neutralization (Supplementary Table S2). At 12 weeks post-MI, the infarct is a mature, cross-linked collagen scar of fixed size, and adult cardiomyocytes do not regenerate, which can explain that appreciable reverse of cardiac remodeling would not occur when rats with the late stage of CHF were treated by a two-week neutralization with GM-CSF. The same results has been reported by our previous work [13], in which renal denervation attenuates SG neuroinflammation, sympathetic activation, and ventricular arrhythmogenesis without the improvement of cardiac function at the late stage of CHF. These observations support a direct action of GM-CSF neutralization on the SG rather than an indirect consequence of improved cardiac performance.

### 3.4. Limitations and Future Perspectives

Several limitations should be considered. Because GM-CSF was neutralized systemically, we cannot determine whether the antibody acted locally within the SG or indirectly by reducing myeloid mobilization and immune trafficking in the periphery. For the same reason, the effects specific to the SG cannot be separated from broader anti-inflammatory actions in the heart, lung, and circulation. Future studies using SG-targeted or cell-specific approaches will be necessary to define the precise neural versus systemic contributions.

From a biochemical perspective, multiple immunoreactive species were detected on the GM-CSF blots across distinct tissue microenvironments; enzymatic deglycosylation would have helped clarify their relationship, and we acknowledge the absence of this assay as a technical limitation of the current work. Relatedly, our cytokine immunoblots detected the precursor forms rather than the mature cleavage products. Mature IL-1β is secreted and rapidly cleared from tissue, so its steady-state concentration in whole-tissue lysate is expected to be low, and it was not detected under our conditions; we therefore draw no inference from its absence and interpret these data as reflecting inflammatory priming rather than active cytokine signaling.

A notable methodological constraint is the reliance on spectral HRV analysis as a proxy for cardiac sympathetic tone, a practice with well-documented limitations in heart failure populations [43]. The LF component and LF/HF ratio are shaped by several influences beyond sympathetic outflow, including baroreflex function, cardiac β-adrenergic receptor sensitivity, post-receptor signal transduction, and parasympathetic modulation [43,44]. Relying on LF and the LF/HF ratio alone to estimate sympathetic activation is therefore weak, particularly in CHF, where post-synaptic β-adrenergic signaling is altered [45]; indeed, LF variability of sympathetic nerve activity is markedly diminished in severe heart failure [46]. Therefore, we directly recorded CSNA, which is not subject to these post-synaptic influences, and combined CSNA and HRV analyses together to confirm cardiac sympathetic activation in this study.

In addition, this study was conducted in male animals, which may limit the generalizability of our findings. Studies incorporating both sexes are warranted to determine whether these effects are sex-specific or universally applicable.

## 4. Methods and Materials

All animal protocols followed the NIH Guide for the Care and Use of Laboratory Animals and received approval from the Institutional Animal Care and Use Committee (IACUC: 24-008-03-FC) at the University of Nebraska Medical Center. To achieve robust and unbiased data, all animals and tissue samples were blinded during experimentation (including the allocation) and data acquisition until statistical analysis.

### 4.1. General Procedures and Experimental Design

Male *Sprague–Dawley* rats (7–8 weeks old, 180–200 g; *n* = 42) were housed in a temperature- and humidity-controlled facility on a 12 h light–dark cycle. Survival surgeries were performed under 2% isoflurane anesthesia, with postoperative analgesia provided via slow-release buprenorphine (1 mg/kg, s.c.). Non-survival terminal procedures were conducted using urethane (800 mg/kg, i.p., Sigma Aldrich, St. Louis, MO, USA) and α-chloralose (40 mg/kg, i.p., Sigma Aldrich, St. Louis, MO, USA). After in vivo experiments were completed, rats were euthanized with i.p. injection of 0.39 mL/kg of Fatal-Plus euthanasia (about 150 mg/kg pentobarbital, DBA Med-Vet International, Mettawa, IL, USA).

Animals were initially randomized into sham-operated or chronic heart failure (CHF) groups. At 12 weeks post-surgery, following echocardiographic confirmation of heart failure, CHF rats were further randomized to receive either a vehicle or a GM-CSF neutralizing antibody (10 µg/kg/day) via a 14-day continuous infusion [47]. Thirteen weeks following myocardial infarction or sham surgery, all animals were implanted with radiotelemetry devices. Twenty-four-hour continuous ECG recordings were obtained from conscious, freely moving rats to assess heart rate variability (HRV). Terminal experiments including cardiac sympathetic nerve activity (CSNA), whole-cell patch-clamp recordings, ELISA, and Western blotting were performed at the end of 14 weeks.

### 4.2. Surgical Induction of Chronic Heart Failure

CHF was established through permanent ligation of the left anterior descending (LAD) coronary artery. Following a left thoracotomy under isoflurane anesthesia, the heart was exteriorized and the left atrial appendage was minimally retracted to expose the proximal LAD. The artery was ligated approximately 2 mm from its origin. Successful occlusion was confirmed by the immediate onset of myocardial blanching and cyanosis in the distal left ventricle. Sham-operated rats underwent an identical surgical exposure and cardiac manipulation without arterial ligation. Development of CHF was verified 12 weeks post-infarction using transthoracic echocardiography to assess structural and functional cardiac parameters [13,38].

### 4.3. Mini-Osmotic Pump Implantation

At 12 weeks post-MI, Alzet mini-osmotic pumps (Model 2002, DURET Corporation, Cupertino, CA, USA) were implanted to deliver either GM-CSF neutralizing antibody (Cat. No. MAB5181, R&D Systems, Minneapolis, MN, USA) or phosphate-buffered saline vehicle. Pumps were filled under aseptic conditions and primed overnight at 37 °C per manufacturer specifications. Under isoflurane anesthesia, the pumps were placed in a subcutaneous pocket created in the mid-scapular region. This system provided a continuous systemic delivery of the antibody at a dose of 10 µg/kg/day for the last two weeks of the study.

### 4.4. ECG Telemeter Implantation for Assessment of Heart Rate Variability

Briefly, a midline laparotomy was performed to position the ECG telemeter (Model TR50B, Millar Instruments, Houston, TX, USA) within the abdominal cavity, where it was secured to the abdominal wall. Bipolar leads were tunneled subcutaneously, with the negative electrode positioned in the upper right pectoralis region and the positive electrode secured near the xiphoid process. Leads were routed in close proximity to minimize signal noise. Following a one-week recovery period, 24 h continuous ECG recordings were obtained from conscious, unrestrained rats using a SmartPad receiver and a PowerLab 8/30 acquisition system (AD Instruments, Colorado Springs, CO, USA). Heart rate variability was analyzed using LabChart 8 software (AD Instruments, Colorado Springs, CO, USA) with the specialized ECG analysis module for high-fidelity detection of R-waves and beat-to-beat intervals. Power spectral analysis was performed on eight 5 min segments of stable recording, one selected from each consecutive 3 h interval across the 24 h period, and the resulting values were averaged. Power spectral density was calculated for LF power (0.2–0.75 Hz), HF power (0.75–2.5 Hz), and the LF/HF ratio as indices of cardiac autonomic balance, including cardiac sympathetic and parasympathetic modulation [13,38].

### 4.5. Recording of the CSNA

A left thoracotomy was performed through the second intercostal space to expose the left stellate ganglion and distally associated cardiac sympathetic nerve (CSN). The CSN was carefully isolated from the surrounding connective tissue and positioned onto a platinum-coated bipolar recording electrode. The nerve–electrode interface was encapsulated using a high-viscosity silicone elastomer (Kwik-Cast; World Precision Instruments, Sarasota, FL, USA). Once the nerve was secured, the distal end was transected to eliminate afferent signals, thereby isolating the efferent cardiac sympathetic discharge. The CSNA was recorded using an Isolated Differential Amplifier (ISO—80; World Precision Instruments, Sarasota, FL, USA). The data was acquired using a PowerLab 8/30 system and LabChart 8 software (AD Instrument, Colorado Springs, CO, USA). Maximal CSNA was obtained after intracardiac injection of 3M KCl and the data was expressed as the percentage of maximum CSNA [48].

### 4.6. Western Blotting

Tissue samples were harvested at the terminal time point, snap-frozen on dry ice, and stored at −80 °C. Protein lysates were prepared by homogenizing the tissue in a modified RIPA lysis buffer (50 mM Tris-HCl, 150 mM NaCl, 50 mM NaF, 2 mM EDTA, 1 mM, 1% NP-40, 1% SDS, and 1 mM PMSF) supplemented with a protease inhibitor cocktail (MedChemExpress (MCE), Monmouth Junction, NJ, USA). Following centrifugation (14,000× *g* at 4 °C), the supernatant was collected, and total protein concentrations were determined via a bicinchoninic acid (BCA) assay. Equal amounts of protein (35–50 µg per lane) were resolved on 12% SDS-PAGE gels and transferred to PVDF membranes using a wet-transfer system. Membranes were blocked for 1 h at room temperature in Intercept Blocking Buffer (LI-COR Biosciences, Lincoln, NE, USA; Part No. 927-70001) and subsequently incubated overnight at 4 °C with primary antibodies (listed in Supplementary Table S3) under constant agitation. Following incubation with appropriate IRDye-conjugated secondary antibodies including IRDye 800CW, IRDye 680RD, and IRDye 680LT (LI-COR Biosciences, Lincoln, NE, USA), immunoreactive bands were visualized using the LI-COR Odyssey DLx imaging platform. Densitometric quantification was performed using ImageJ software (version 1.54m NIH, Bethesda, MA, USA), with protein expression levels normalized to β-actin or GAPDH to ensure consistent loading. The full blots for representative cropped images were provided in Supplementary Figures S2–S7.

### 4.7. Enzyme-Linked Immunosorbent Assay (ELISA)

At the terminal time point, whole blood was collected by cardiac puncture into serum separator tubes. Samples were allowed to clot at room temperature. Following centrifugation (2000× *g* for 15 min), the serum fraction was aliquoted and stored at −80 °C. Circulating levels of GM-CSF were quantified using a commercially available rat-specific ELISA kit (Cat. No. DY518; R&D Systems, Minneapolis, MN, USA) in accordance with the manufacturer’s instructions. All samples and standards were run in duplicate, and the absorbance was measured at 450 nm using a microplate reader (Infinite 200, Tecan Group Ltd., Männedorf, Switzerland).

### 4.8. Isolation of Cardiac Sympathetic Post-Ganglionic Neurons in the SGs and Whole-Cell Patch-Clamp Recording for Ca^2+^ Currents and APs

After in vivo experiments were performed, bilateral SGs were exposed and removed quickly. SG neurons were isolated by a two-step enzymatic digestion protocol as described previously [9,38,39]. Briefly, the isolated SGs were placed in ice-cold modified Tyrode’s solution (mM): 140 NaCl, 5 KCl, 10 HEPES, 5 glucose. The SGs were micro-dissected to remove the connective sheath, minced into small fragments, and incubated in modified Tyrode’s solution containing 0.1% collagenase and 0.1% trypsin for 30 min at 37 °C. The tissue was subsequently transferred to a second solution containing 0.2% collagenase and 0.5% bovine serum albumin (BSA) for an additional 30 min incubation. Finally, the dissociated CSP neurons were cultured in a humidified environment (95% air/5%) at 37 °C for 4–8 h prior to electrophysiological recording.

Voltage-gated Ca^2+^ currents and APs were recorded by the whole-cell patch-clamp technique using Axopatch 200B patch-clamp amplifier (Axon Instruments, Colorado Springs, CO, USA). The resistance of the patch pipette was 4–6 MΩ when the pipette was filled with the following solution (in mM): 120 CsCl, 1 CaCl_2_, 40 HEPES, 11 EGTA, 4 MgATP, 0.3 Tris-GTP, 14 creatine phosphate, and 0.1 leupeptin (pH 7.3; 305 mosM). The extracellular solution consisted of (in mM): 140 TEA-Cl, 5 BaCl_2_, 1 MgCl_2_, 10 HEPES, 0.001 TTX, 2 4-aminopyridine (4-AP), and 10 glucose (pH 7.4; 310 mosM). Series resistance of 5–13 MΩ was electronically compensated 30–80%. Junction potential was calculated to be +7.9 mV using pCLAMP 10.2 software, and all values of membrane potential given throughout were corrected using this value. Current traces were sampled at 10 kHz and filtered at 5 kHz. The holding potential was −80 mV and current–voltage (I−V) relationships were elicited by 5 mV step increments to potentials between −60 mV and 60 mV for 500 ms. Peak currents were measured for each test potential, and current density was calculated by dividing peak current by cell membrane capacitance (25.7 ± 1.7 pF in sham, 28.8 ± 2.1 pF in CHF, and 28.6 ± 1.7 pF in CHF + GM-CSF Ab neurons, *p* > 0.05).

In current-clamp experiments, AP was elicited by a ramp current injection of 0–100 pA and the current threshold-inducing AP was measured at the beginning of the 1st AP. Frequency of APs was measured in a 1 s current clamp at 100 pA. The patch pipette solution was composed of (in mM): 105 K-aspartate, 20 KCl, 1 CaCl_2_, 5 MgATP, 10 HEPES, 10 EGTA, and 25 glucose (pH 7.2; 320 mOsm/L). The bath solution was composed of (in mM): 140 NaCl, 5.4 KCl, 0.5 MgCl_2_, 2.5 CaCl_2_, 5.5 HEPES, 11 glucose, and 10 sucrose (pH 7.4; 330 mOsm/L). Junction potential was calculated to be +12.3 mV, and membrane potential was corrected using this value. The pCLAMP 10.2 program (Axon Instruments, Colorado Springs, CO, USA) was used for data acquisition and analysis. All experiments were performed at room temperature (22–24 °C).

### 4.9. Statistical Analysis

Quantitative data were analyzed and visualized using GraphPad Prism 10.5.1 (GraphPad Software, San Diego, CA, USA). Data distribution and normality were assessed using the Shapiro–Wilk test and visual inspection of Q-Q plots. For normally distributed data with equal variance (confirmed by Bartlett’s test), group differences were evaluated via one-way ANOVA followed by Tukey’s post hoc test. In cases of heteroscedasticity, a Welch ANOVA with Dunnett’s T3 post hoc test was applied. For non-parametric data, the Kruskal–Wallis test followed by Dunn’s post hoc test was utilized. Results are expressed as mean ± SEM. Statistical significance was defined as *p* < 0.05.

## 5. Conclusions

This study identified GM-CSF as a key regulator of the neuroimmune crosstalk within the SG that sustained pathological sympathetic overactivation in CHF. Systemic neutralization of GM-CSF attenuated inflammation in the SG, partially normalized Ca^2+^ currents, and lowered neuronal firing patterns, alongside more stable cardiac sympathetic activity.

## Data Availability

The original contributions presented in this study are included in the article/Supplementary Materials. Further inquiries can be directed to the corresponding author.

## Supplementary Materials

The following supporting information can be downloaded at https://www.mdpi.com/article/10.3390/ijms27156876/s1.
