# Supplementary material for: Systemic Neutralization of Granulocyte–Macrophage Colony-Stimulating Factor Attenuates Stellate Ganglion Neuroinflammation and Cardiac Sympathetic Overactivation in Chronic Heart Failure in Rats
Source: Int J Mol Sci. 2026 Jul 31;27(15):6876. doi: 10.3390/ijms27156876 (PMC13466555; doi:10.3390/ijms27156876)
Supplement: Supplementary file 1 [file ijms-27-06876-s001.zip › ijms-4422934-supplementary.pdf]

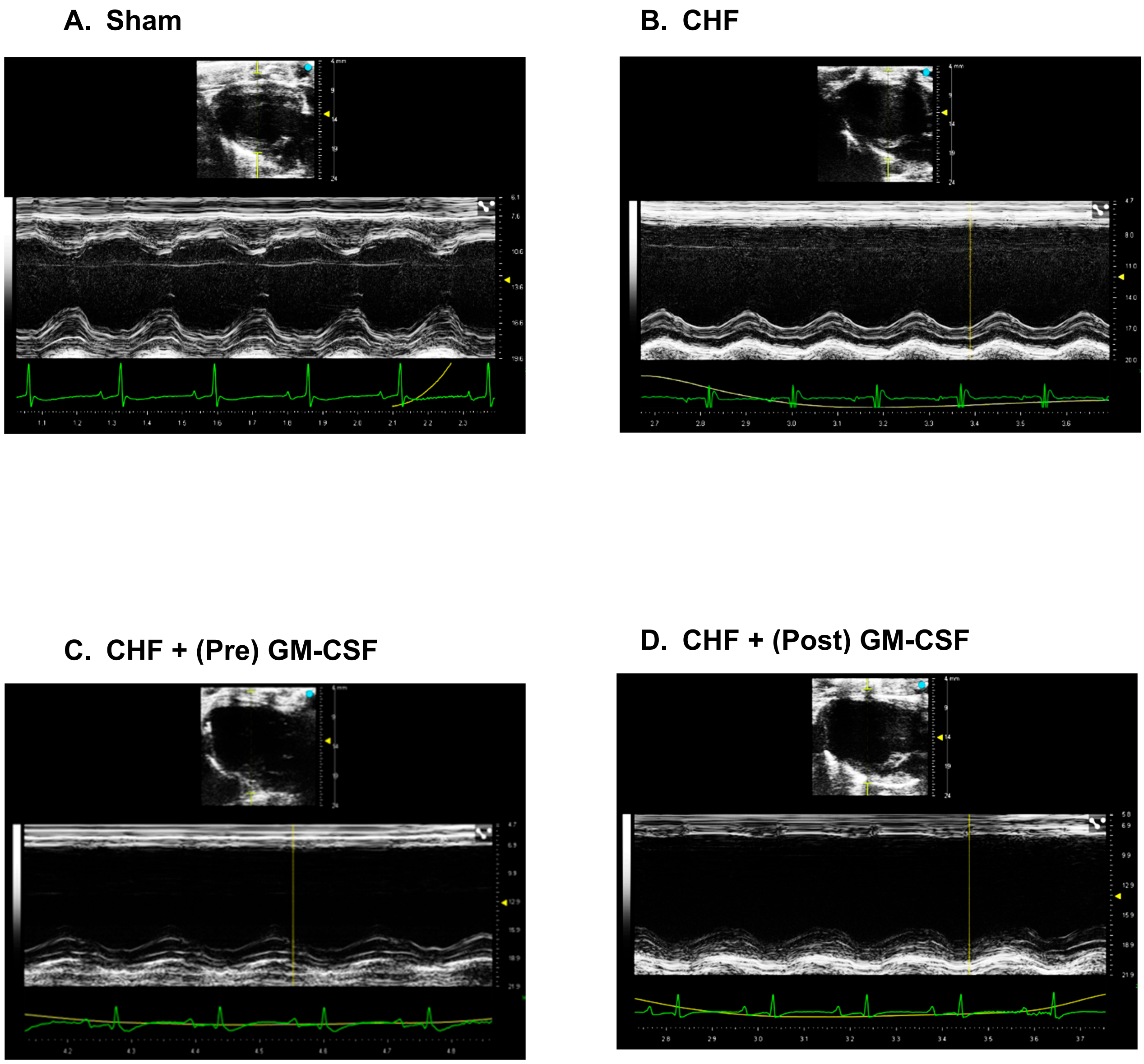

Supplementary Figure S1. Representative B-mode (upper) and M-mode (bottom) echocardiographic images in Sham, CHF, CHF+ (Pre) GM-CSF Ab, CHF+ (Post) GM-CSF Ab groups.

Supplementary Table S1. Echocardiography Data for CHF phenotype confirmation at 12 weeks post-surgery.

Parameters	Sham (n=6)	CHF (n=6)	CHF + (Pre) GM-CSF Ab (n=6)
LVEF (%)	64.4±1.03	31.7±2.05*	31.8±1.57*
LVFS (%)	36.7±0.74	16.0±1.14*	16.0±0.88*
LVDs (mm)	5.33±0.14	8.91±0.29*	8.71±0.08*
LVDd (mm)	8.47±0.15	10.7±0.26*	10.4±0.12*

Data are presented as Mean ± SEM. Statistical significance was determined by one-way ANOVA followed by Tukey's post-hoc test. Abbreviations: LVEF, left ventricular ejection fraction; LVFS, left ventricular fractional shortening; LVDs, left ventricular dimension during systole; LVDd, Left ventricular dimension during diastole. * $p < 0.05$ vs Sham.

Supplementary Table S2. Comparison of cardiac function data before and after GM-CSF neutralization.

Parameters	CHF + (Pre) GM-CSF Ab (n=6)	CHF + (Post) GM-CSF Ab (n=6)
LVEF (%)	31.8±1.57	32.1±1.15^{ns}
LVFS (%)	16.0±0.88	16.1±0.63 ^{ns}
LVDs (mm)	8.71±0.08	8.51±0.19 ^{ns}
LVDd (mm)	10.4±0.12	10.2±0.20 ^{ns}

Data are presented as Mean ± SEM. Statistical significance was determined by Paired T-test. Abbreviations: LVEF, left ventricular ejection fraction; LVFS, left ventricular fractional shortening; LVDs, left ventricular dimension during systole; LVDd, Left ventricular dimension during diastole. (ns = not significant vs. CHF + (Pre) GMCSF Ab).

Supplementary Table S3. Specifications of primary antibodies used for western blot.

Target Antigen	Host	Catalogue No.	Company	Dilution
β-actin	Mouse	sc-47778	Santa Cruz Biotechnology	1:1000
GAPDH	Mouse	sc-32233	Santa Cruz Biotechnology	1:1000
GM-CSF	Goat	PA1-28021	Invitrogen	1:250
IBA1	Rabbit	ab178846	Abcam	1:1000
Pro IL-1β	Goat	PA5-46956	Invitrogen	1:250
TNF-α	Rabbit	17590-1-AP	Proteintech	1:500

Abbreviations: β-actin, beta-actin; GAPDH, glyceraldehyde 3-phosphate dehydrogenase; GM-CSF, granulocyte-macrophage colony-stimulating factor; IBA1, ionized calcium-binding adapter molecule 1; Pro IL-1β, Pro interleukin 1 beta; TNF-α, tumor necrosis factor alpha.

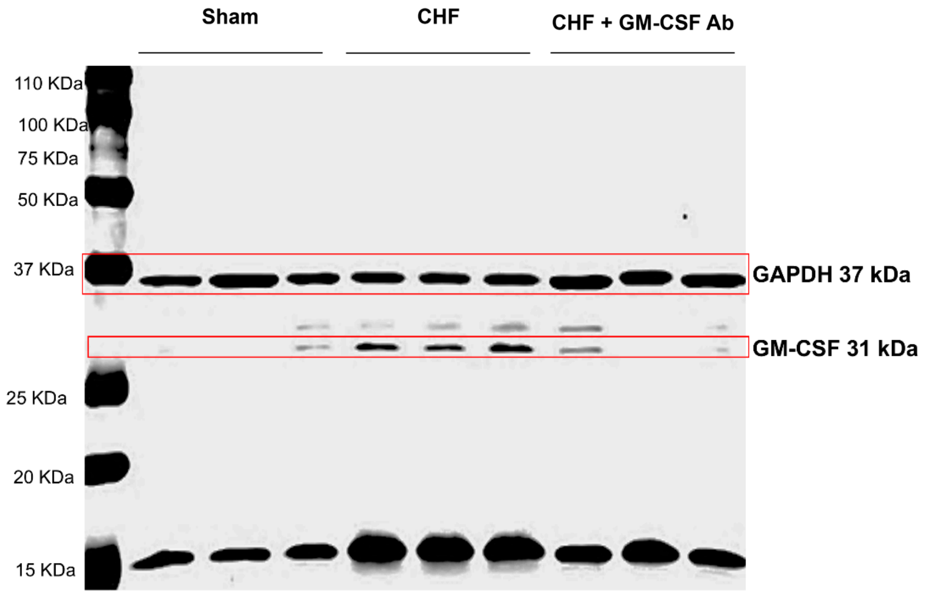

Supplementary Figure S2. The full blot for representative cropped image presented in Figure 1a. GAPDH (37 kDa) and GM-CSF (31 kDa) expression in stellate ganglia in Sham, CHF and CHF+GM-CSF Ab groups. (GM-CSF was quantified at ~31kDa as this immunoreactive specie was detected consistently across heart (fig. S6), lung (fig. S7) and stellate ganglia (fig. S2).

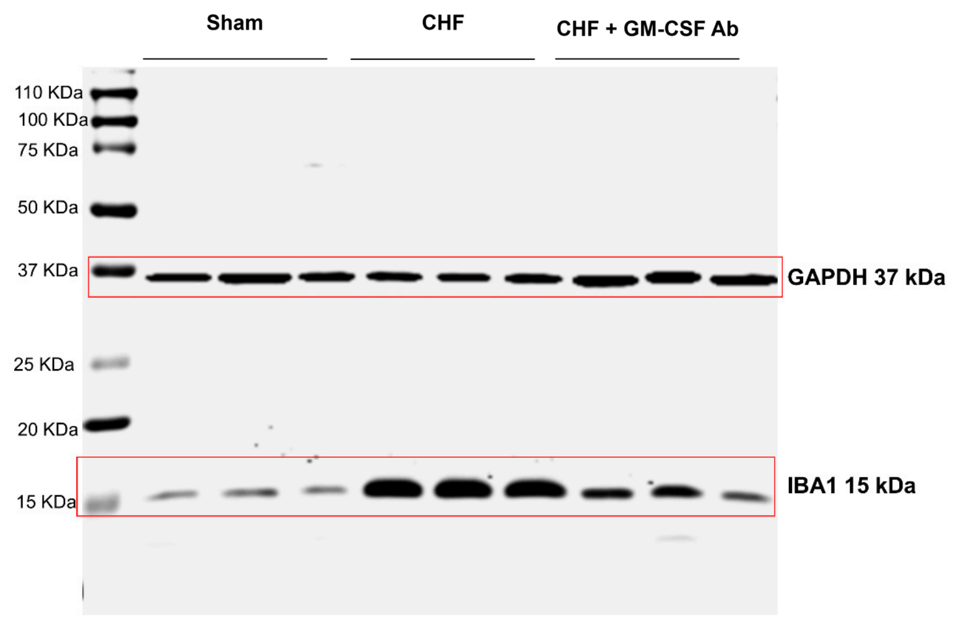

Supplementary Figure S3. The full blot for representative cropped image presented in Figure 1b. GAPDH (37 kDa) and IBA1 (15 kDa) expression in stellate ganglia in Sham, CHF and CHF+GM-CSF Ab groups.

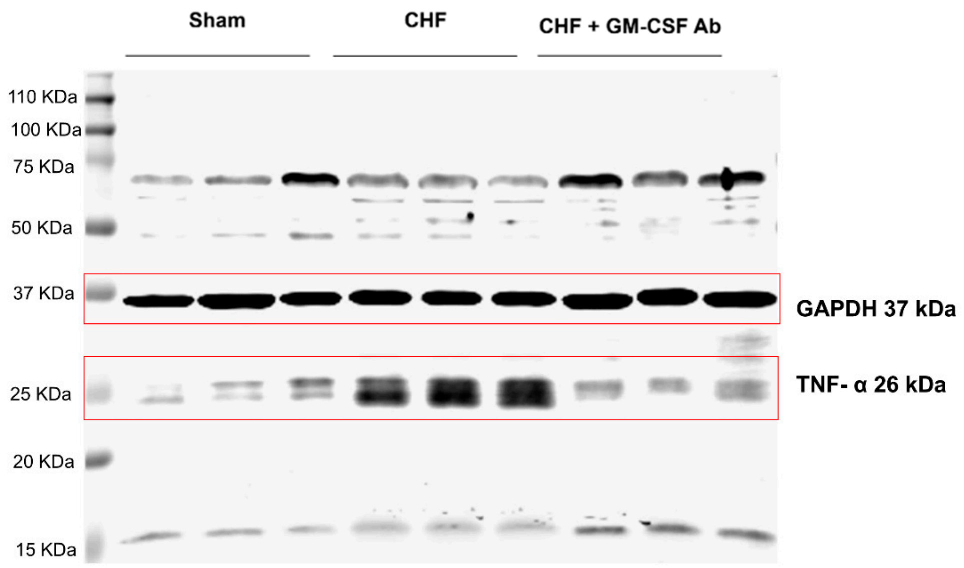

Supplementary Figure S4. The full blot for representative cropped image presented in Figure 1c. GAPDH (37 kDa) and TNF-α (26 kDa) expression in stellate ganglia in Sham, CHF and CHF+GM-CSF Ab groups.

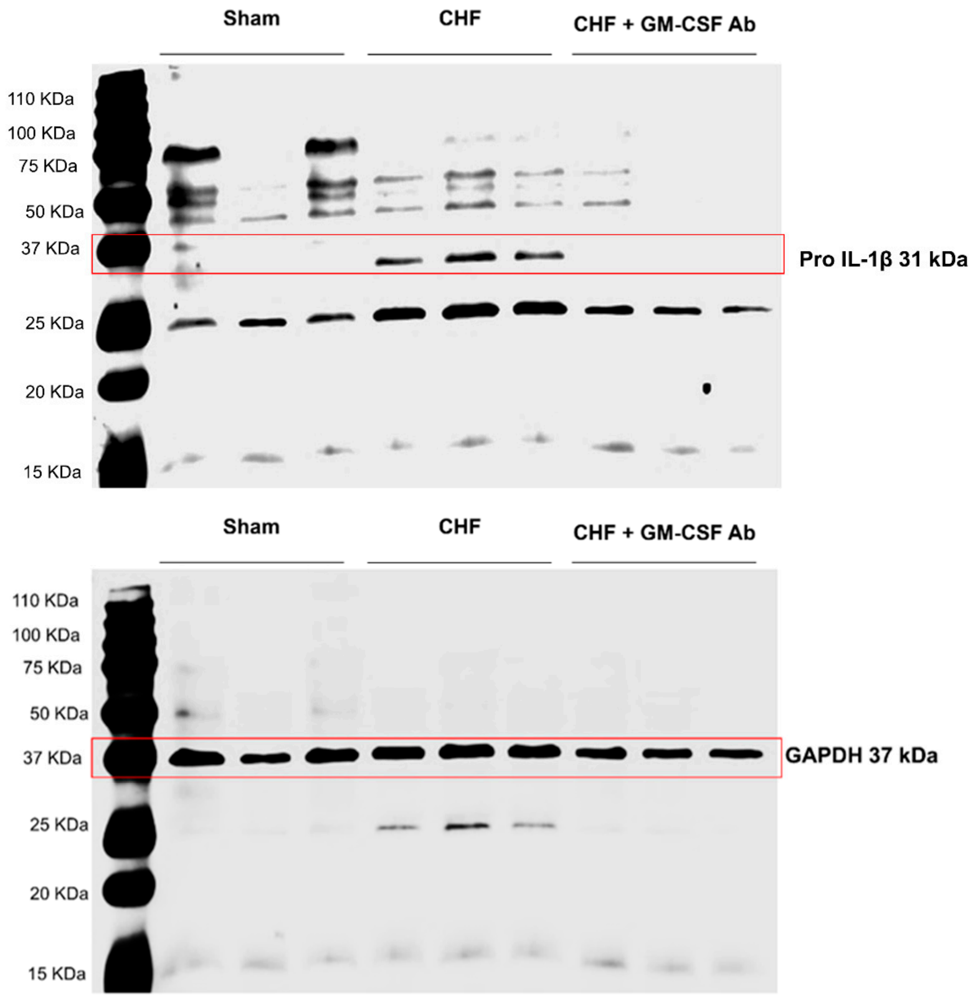

Supplementary Figure S5. The full blots for representative cropped image presented in Figure 1d. GAPDH (37 kDa) and Pro IL-1β (31 kDa) expression in stellate ganglia in Sham, CHF and CHF+GM-CSF Ab groups. The membrane was stripped before probing for GAPDH.

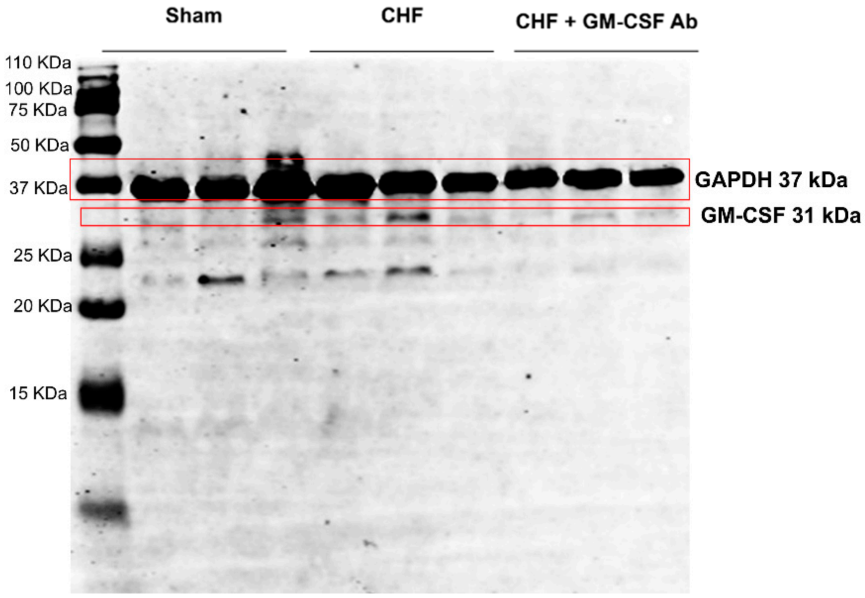

Supplementary Figure S6. The full blot for representative cropped image presented in Figure 2a. GAPDH (37 kDa) and GM-CSF (31 kDa) expression in the heart tissue in Sham, CHF and CHF+GM-CSF Ab groups.

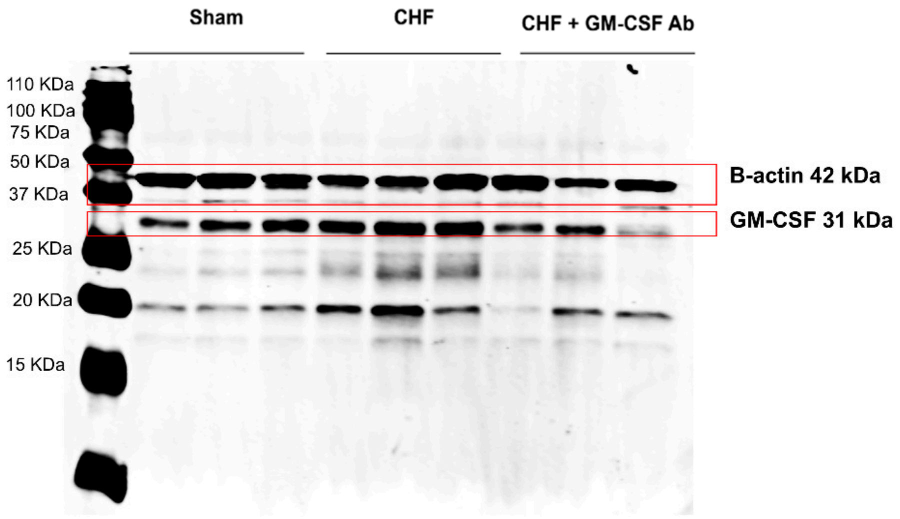

Supplementary Figure S7. The full blot for representative cropped image presented in Figure 2b. β-actin (42 kDa) and GM-CSF (31 kDa) expression in the lung tissue in Sham, CHF and CHF+GM-CSF Ab groups.
